# Supplementary figures and images for: A Selective Inhibitor of Cardiac Troponin I Phosphorylation by Delta Protein Kinase C (δPKC) as a Treatment for Ischemia-Reperfusion Injury
Source: Pharmaceuticals (Basel). 2022 Feb 22;15(3):271. doi: 10.3390/ph15030271 (PMC8950820; doi:10.3390/ph15030271)

Figure S1

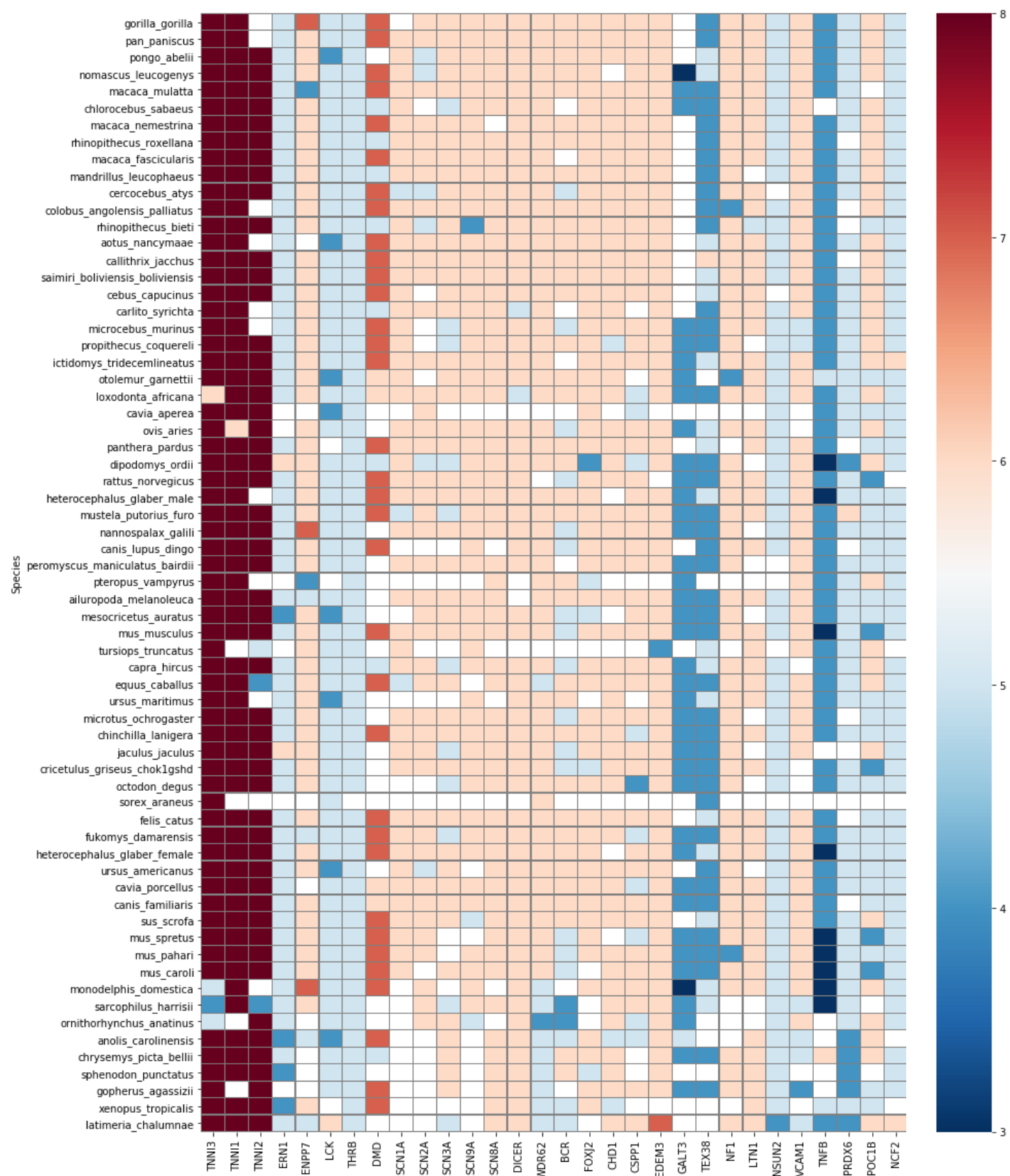

Supplement: Supplementary file 1 [file pharmaceuticals-15-00271-s001.zip › pharmaceuticals-1589061-supplementary.pdf]
